# Comparative Analysis of Bacterial Communities in *Lutzomyia ayacuchensis* Populations with Different Vector Competence to *Leishmania* Parasites in Ecuador and Peru

**DOI:** 10.3390/microorganisms9010068

**Published:** 2020-12-29

**Authors:** Ahmed Tabbabi, Shinya Watanabe, Daiki Mizushima, Abraham G. Caceres, Eduardo A Gomez, Daisuke S. Yamamoto, Longzhu Cui, Yoshihisa Hashiguchi, Hirotomo Kato

**Affiliations:** 1 Division of Medical Zoology, Department of Infection and Immunity, Jichi Medical University, Tochigi 329-0498, Japan; tabbabiahmed@gmail.com (A.T.); dmizushima@jichi.ac.jp (D.M.); daisukey@jichi.ac.jp (D.S.Y.); 2 Division of Bacteriology, Department of Infection and Immunity, Jichi Medical University, Tochigi 329-0498, Japan; swatanabe@jichi.ac.jp (S.W.); longzhu@jichi.ac.jp (L.C.); 3 Sección de Entomología, Instituto de Medicina Tropical “Daniel A. Carrión” y Departamento Académico de Microbiología Médica, Facultad de Medicina Humana, Universidad Nacional Mayor de San Marcos, Lima 15081, Peru; acaceres31@hotmail.com; 4 Laboratorio de Entomología, Instituto Nacional de Salud, Lima 15081, Peru; 5 Departamento de Parasitologia y Medicina Tropical, Facultad de Ciencias Medicas, Universidad Catolica de Santiago de Guayaquil, Guayaquil 090615, Ecuador; egolandires@yahoo.es; 6 Department of Parasitology, Kochi Medical School, Kochi University, Kochi 783-8505, Japan; yhashiguchi42@yahoo.co.jp

**Keywords:** *Lutzomyia ayacuchensis*, microbiota, *Leishmania*, Peru, Ecuador

## Abstract

Differences in the gut microbial content of *Lutzomyia (Lu.) ayacuchensis*, a primary vector of Andean-type cutaneous leishmaniasis in Ecuador and Peru, may influence the susceptibility of these sand flies to infection by *Leishmania*. As a first step toward addressing this hypothesis, a comparative analysis of bacterial and fungal compositions from *Lu. ayacuchensis* populations with differential susceptibilities to *Leishmania* was performed. Bacterial 16S rRNA gene amplification and Illumina MiSeq sequencing approaches were used to characterize the bacterial composition in wild-caught populations from the Andean areas of Ecuador and southern Peru at which the sand fly species transmit *Leishmania* (*Leishmania*) *mexicana* and *Leishmania* (*Viannia*) *peruviana*, respectively, and a population from the northern Peruvian Andes at which the transmission of *Leishmania* by *Lu. ayacuchensis* has not been reported. In the present study, 59 genera were identified, 21 of which were widely identified and comprised more than 95% of all bacteria. Of the 21 dominant bacterial genera identified in the sand flies collected, 10 genera had never been detected in field sand flies. The Ecuador and southern Peru populations each comprised individuals of particular genera, while overlap was clearly observed between microbes isolated from different sites, such as the number of soil organisms. Similarly, *Corynebacterium* and *Micrococcus* were slightly more dominant bacterial genera in the southern Peru population, while *Ochrobactrum* was the most frequently isolated from other populations. On the other hand, fungi were only found in the southern Peru population and dominated by the *Papiliotrema* genus. These results suggest that variation in the insect gut microbiota may be elucidated by the ecological diversity of sand flies in Peru and Ecuador, which may influence susceptibility to *Leishmania* infection. The present study provides key insights for understanding the role of the microbiota during the course of *L.* (*L.*) *mexicana* and *L. (V.) peruviana* infections in this important vector.

## 1. Introduction

Similar to other living organisms, insects are intimately associated with bacteria at all stages of their lives, suggesting that the resident microbiota acts as a driving force by affecting several aspects of host biology [1,2,3,4,5]. Hematophagous phlebotomine sand flies (Diptera: Psychodidae) are responsible for the transmission of protozoan parasites that cause leishmaniasis [6]. Males and females both feed on natural sugar sources, such as the sap of plants or honeydew, through which they may acquire plant bacteria [7], and adult females also feed on the blood of a wide range of hosts, including humans, in order to develop eggs and reproduce. During larval development, sand fly larvae are exposed to a wide variety of soil bacteria and other microorganisms that have the ability to colonize the insect gut. Previous studies showed that most of these larval-stage bacteria undergo biodegradation during the pupal stage and the bacterial load suddenly and significantly decreases after an adult’s emergence [8,9]. Additionally, female sand flies acquire *Leishmania* parasites when they feed on an infected mammalian host in search of a blood meal, and the *Leishmania* developmental life cycle within the sand fly vector occurs exclusively in the mid- and hindgut with the presence of symbiotic bacteria; therefore, possible bacteria–parasite interactions occur between the microbial community of the gut and parasite [10,11]. The bacterial composition of the gut was previously reported to either enhance or inhibit parasite activity depending on the species of bacteria, and, thus, has the potential to alter vector competence [8,11,12,13,14,15]. The diversity of the gut bacterial community may be related to the geographical distribution of sand flies, and each geographically distinct population may have different bacterial structures [16]. As a critical aspect of sand fly biology and ecology, several surveys of microflora have been conducted on Old and New World sand flies [8,9,10,16,17,18,19,20,21,22,23]. Despite the public health importance of *Lutzomyia (Lu.) ayacuchensis*, their microbiota composition has not yet been elucidated.

*Lu. ayacuchensis* is a principal vector of cutaneous leishmaniasis in the Andean highlands of Ecuador and Peru, with a wide geographic distribution [24,25,26,27,28,29]. This species is the proven vector of *Leishmania (Leishmania) mexicana* in the Ecuadorian Andes [24,26,27,28,29,30]; however, the same species is responsible for the transmission of *L. (Viannia) peruviana* in the Andean areas of southern Peru [25]. *Lu. ayacuchensis* also occurs in the northern Peruvian Andes, at which the transmission of *Leishmania* has not been reported, although the potential transmission of *Leishmania* by this population cannot be completely excluded. Molecular markers were recently evaluated to assess genetic divergence among *Lu. ayacuchensis* populations with different levels of vector competence [31]. Recent studies on several insect systems indicated that both heritable symbionts and environmentally-acquired commensal microbiota-induced phenotypes affect an insect host’s capacity to transmit pathogens [4]. Based on this information, we analyzed the potential of the bacterial communities of *Lu. ayacuchensis* to play a significant role in successful *Leishmania* infection, which may, thus, influence vector competence. We investigated differences in bacterial and fungal compositions among three wild-caught *Lu. ayacuchensis* groups with contrasting susceptibilities to *Leishmania* infection. Although the conflicting capacities of these different groups to transmit different *Leishmania* species were previously shown to be associated with complex genetic factors [31], the role of the existing midgut bacterial microbiota in the observed phenotypic variability in the susceptibility phenotype remains unknown.

## 2. Materials and Methods

### 2.1. Study Area and Sand Fly Collection

**Figure 1:**
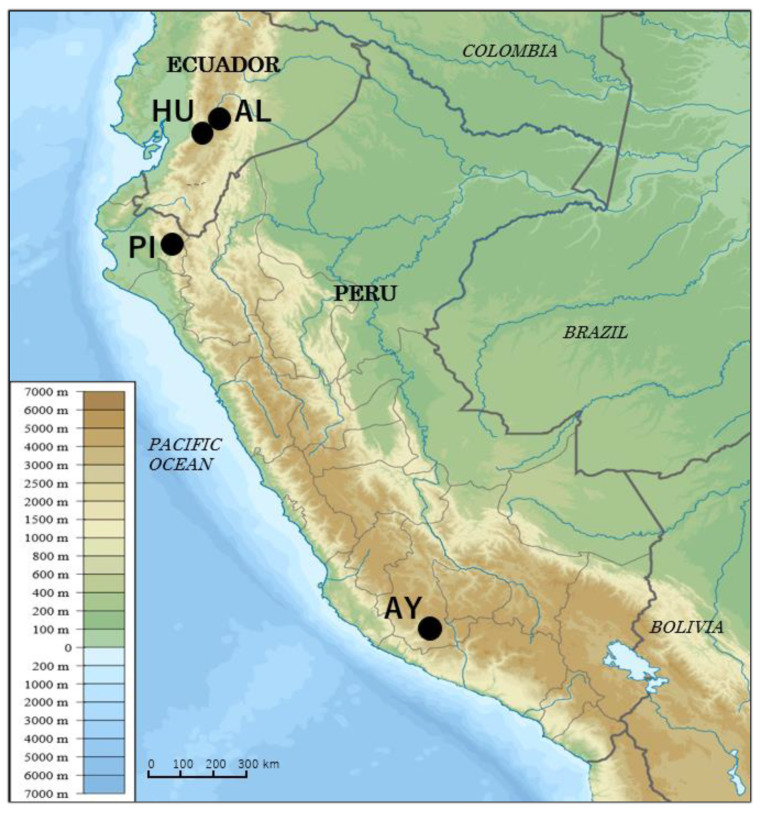
Map of Ecuador and Peru showing geographic locations at which *Lutzomyia ayacuchensis* were collected. AL, Alausi (2365 m altitude); HU, Huigra (1568 m altitude); PI, Huancabamba, Department of Piura (1938 m altitude); AY, Sancos, Department of Ayacucho (2819 m altitude). (Adapted from a map available at https://commons.wikimedia.org/wiki/File%3APeru_physical_map.svg).

Adult sand flies were captured in 3 different districts located in the Andean areas of Ecuador and Peru [Ecuador: Alausi (2365 m above sea level) and Huigra (1568 m asl) in the province of Chimborazo; Northern Peru: Huancabamba (1938 m asl), Department of Piura; and Southern Peru: Sancos (2819 m asl), Department of Ayacucho] [31] (Figure 1). Specimens were captured using the Centers for Disease Control (CDC) miniature light traps (John W. Hock Co., Gainesville, FL, USA), which were set before sunset and collected the following morning. Sand flies were also captured between 18:30 and 21:00 by protected human bait and between 18:00 and 22:00 using Shannon traps. Individual sand flies were surface sterilized in 70% ethanol and dried well before DNA extraction.

### 2.2. Species Identification and Phylogenetic Analysis

The specimens of *Lu. ayacuchensis* collected were sorted by morphological keys and confirmed by sequencing the mitochondrial cytochrome oxidase I (COI) gene, as reported previously [31]. Sequences were aligned with CLUSTAL W software [32], and the construction of the phylogenetic tree from the aligned sequences was performed using the Maximum Likelihood (ML) method with the distance algorithms available in the MEGA package (Molecular Evolutionary Genetics Analysis) version 5.2 [33].

### 2.3. DNA Extraction

Ethanol-fixed sand flies were individually suspended in 500 μL of saline solution or PBS buffer. One gram of 0.1 mm-diameter zirconia/silica beads (BioSpec Products, Bartlesville, OK, USA) was added to the extraction tubes to facilitate the mechanical lysis of microbial cells using the beads crusher μT-12 (Taitec, Saitama, Japan) [34]. Lysed cells were spun down, and 400 μL of the supernatant was collected for DNA isolation. Genomic DNA was purified from each individual sand fly using a ReliaPrep DNA Clean-up and Concentration System kit (Promega Corporation, Madison, WI, USA), as described by the manufacturer.

### 2.4. Bacterial 16S rDNA Gene Amplification and Illumina MiSeq Sequencing

The variable region (V3-V4) of the 16S rDNA gene was amplified separately using 2 PCR steps. In the first round, PCR amplification was performed immediately after DNA extraction, using primers targeting the V3 and V4 regions of the 16S rDNA gene for bacteria. The hypervariable regions V3-V4 of the 16S rDNA gene were amplified using the following primers: forward primer: (5′TCGTCGGCAGCGTCAGATGTGTATAAGAGACAG CCTACGGGNGGCWGCAG-3′) and reverse primer (5′-GTCTCGTGGGCTCGGAGATGTGTATAAGA GACAGGACTACHVGGGTATCTAATCC-3′). These regions were recommended by the Illumina protocol manual [35], and yielded high-quality sequence data, as previously reported [36]. PCR amplification was performed with 35 cycles of denaturation (95 °C, 30 s), annealing (55 °C, 30 s), and polymerization (72 °C, 30 s) using AmpliTaq Gold 360 DNA polymerase. Each 1.5 μL portion of the PCR product was re-amplified with the index primers used to generate amplicon libraries for Illumina sequencing [35]. Amplification success was verified on a 1.5% agarose gel. Sequencing was performed using the Illumina MiSeq platform with MiSeq reagent kit version 3 (Illumina, Inc., San Diego, CA, USA).

### 2.5. Fungal ITS1 Gene Amplification and Illumina MiSeq Sequencing

The fungal ITS1 region was amplified separately using 2 PCR steps as described above for bacterial DNA. Considering the different types of biases (specificity to fungi, mismatches, length, and taxonomy), different primer combinations targeting different parts of the ITS region were analyzed in parallel, as recommended by the Illumina protocol manual [37]. ITS regions were sequenced by the Illumina Miseq platform and MiSeq reagent kit version 3 (Illumina).

### 2.6. Data Processing and Analysis

The reads generated from the MiSeq platform (2 × 301 bp, paired-end format) were exported as fastq files for importation into Quantitative Insights Into Microbial Ecology 2 (QIIME2) (version 2020.2.0) [38]. Paired-end reads were trimmed and merged by the DADA2 program in the QIIME2 Plugin. Sequences were clustered in operational taxonomic units (OTUs) by the QIIME2 program. OTUs were annotated based on the dataset of SILVA version 132 [39] with ≥99% sequence identity.

To assess the diversity of sand fly populations in different areas, 2 alpha (Faith’s Phylogenetic Diversity and Pielou’s evenness) and beta (distance matrix) diversity indices were used: Faith’s PD [40], which was the phylogenetic analog of taxon richness and was expressed as the number of tree units found in a sample; Pielou’s evenness [41], which was defined as a measure of biodiversity that quantifies how equal the members of a community are in terms of their numbers; and Beta diversity metrics, which provided a measure of the degree that samples differed from one another using the number of species shared between communities and reveal aspects of microbial ecology that were not apparent based on the composition of individual samples [42,43]. Alpha and beta diversities were analyzed by QIIME2 with a sampling depth of 400. The group significance of alpha and beta diversity indexes was calculated with QIIME2 plugins using the Kruskal–Wallis test and a permutational multivariate analysis of variance (PERMANOVA), respectively. A *p*-value of less than 0.05 was considered to be significant.

## 3. Results

### 3.1. Analysis of the Lu. ayacuchensis COI Gene

**Figure 2:**
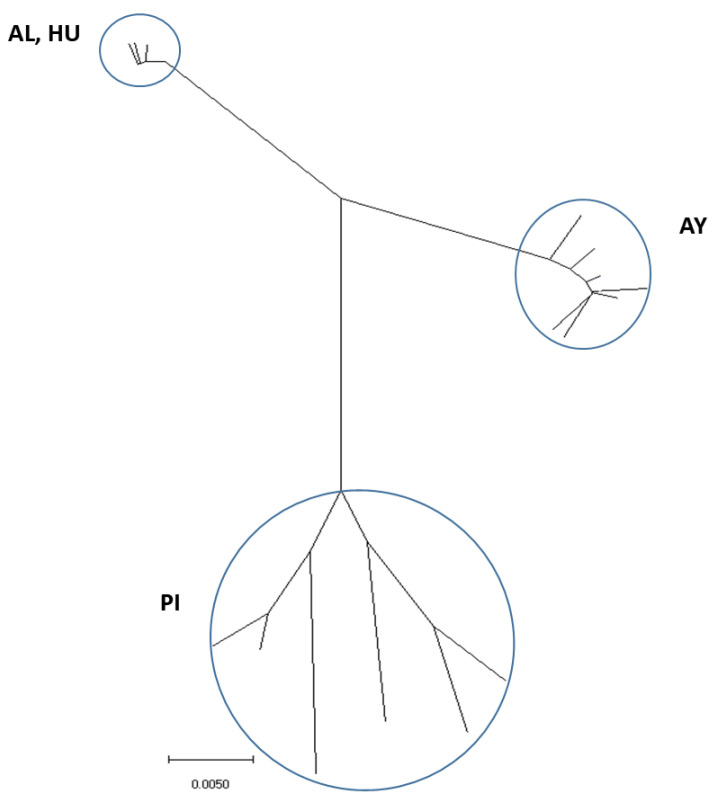
Phylogenetic tree of cytochrome oxidase I sequences among *Lutzomyia ayacuchensis* populations. The scale bar represents 0.005% divergence. AL, Alausi; HU, Huigra; PI, Piura; AY, Ayacucho.

The COI fragment was successfully amplified from each of the 34 specimens from the Andean areas of Ecuador (AL, HU) and southern Peru (AY) at which the sand fly species transmit *L. (L.) mexicana* and *L. (V.) peruviana*, respectively, and the population from the northern Peruvian Andes (PI), at which the transmission of *Leishmania* by *Lu. ayacuchensis* has not been reported. The sequences of 628 bp fragments were aligned and the phylogenetic tree was constructed from the aligned sequences. Clear genetic divergence was noted between populations of *Lu. ayacuchensis* with different vector competence. Populations from Ecuador (AL, HU) clearly consisted of distinct clusters from southern Peru (AY), and the two groups were separated from those from northern Peru (PI) (Figure 2), which is consistent with previous findings [31].

### 3.2. Microbiome Profile of Field-Collected Lu. ayacuchensis

**Figure 3:**
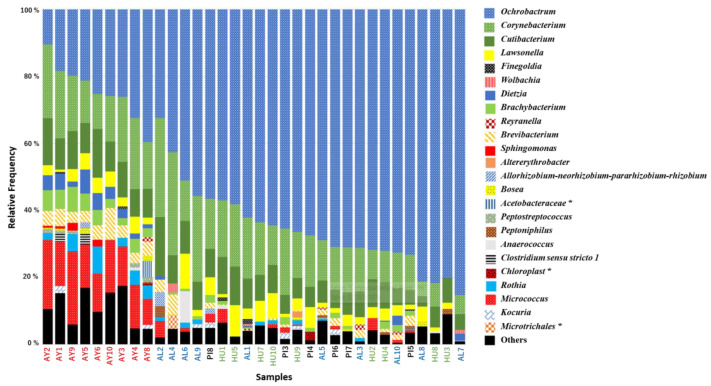
The bacterial genus composition of different *Lutzomyia ayacuchensis* populations. Ecuador (AL, Alausi; HU, Huigra); northern Peru (PI, Piura); southern Peru (AY, Ayacucho). The asterisk indicates other-level classifications when Qiime2 failed to provide the genus level.

The bacterial diversity of wild-caught populations of *Lu. ayacuchensis* adults was investigated using high-throughput sequencing of the 16S rRNA gene. To ensure the highest quality sequencing data, 34 (AL: 10, HU: 9, PI: 6, and AY: 9) out of the 59 analyzed female samples were selected and used in the data analysis of the present study. Taxonomic compositions were also compared between the three groups at the genus level. Fifty-nine genera were identified, of which 21 were widely identified and comprised more than 95% of all bacteria. The latter genera were taxonomically assigned to 15 families of 10 orders in 3 classes and 3 phyla, with a few unclassified taxa at different ranks. Among the 21 dominant bacterial genera identified in the sand flies collected (Figure 3), 10 had never been detected in field sand flies (*Lawsonella*, *Rothia*, *Dietzia*, *Anaerococcus*, *Sphingomonas*, *Peptoniphilus*, *Rayranella*, *Finegoldia*, *Peptostreptococcus*, and *Altereythrobacter*).

**Figure 4:**
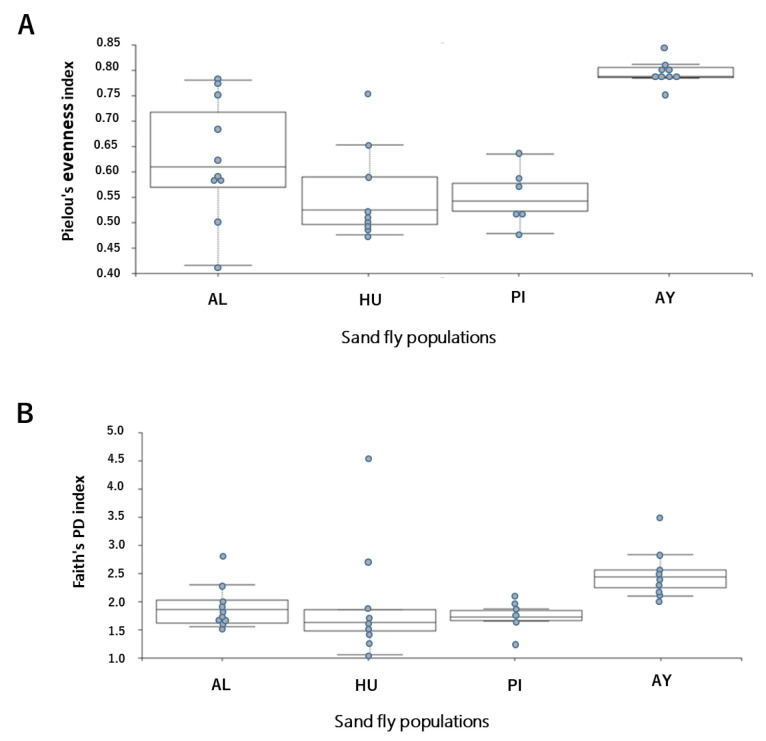
Box plots of the alpha diversity measure using Pielou’s evenness index (**A**) and Faith’s phylogenetic diversity index (**B**) in *Lutzomyia ayacuchensis* populations. Ecuador (AL, Alausi; HU, Huigra); northern Peru (PI, Piura); southern Peru (AY, Ayacucho).

**Figure 5:**
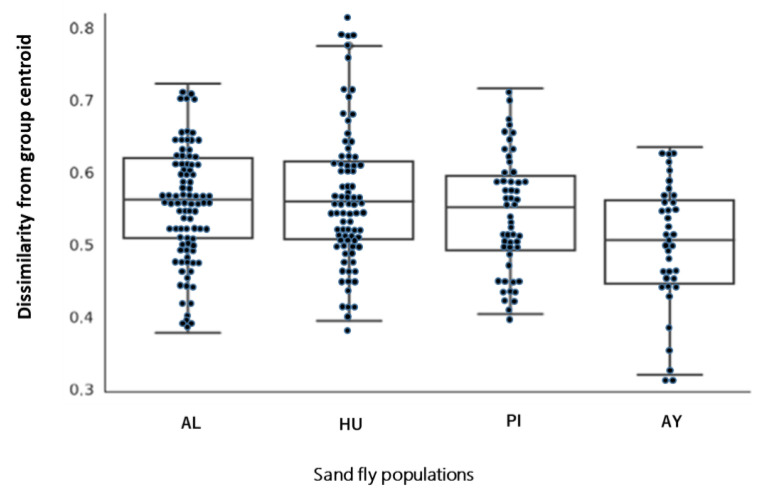
Qiime2 diversity beta- group- significance in *Lutzomyia ayacuchensis* populations. Ecuador (AL (*n* = 90), Alausi; HU (*n* = 81), Huigra)); northern Peru (PI (*n* = 54), Piura); southern Peru (AY (*n* = 36), Ayacucho).

The detailed structure and taxonomy of the microbiome are shown in Figure 3. The alpha diversity index was significantly higher in the southern Peru population (AY) (*p* < 0.05), which may have been due to exposure to several bacterial sources (Figure 4A,B). Similarly, the components of taxonomic beta diversity showed more complex patterns in the southern Peru population (AY) (*p* < 0.05) than in the other populations (Figure 5; Figure S1). Each of Ecuador and southern Peru populations comprised individuals of particular genera, while overlap was clearly observed between microbes isolated from different areas, including a number of soil and environmental organisms, such as species of *Micrococcus* and *Corynebacterium*. It is also important to note that all sand flies collected from different sites of each population harbored similar microbes, suggesting a strong correlation between the sand fly-microbial association and the environment in which they reside. Two bacterial genera: *Clostridium* and *Peptostreptococcus* were only found in the southern Peru population (AY). On the other hand, *Altererythrobacter* and *Wolbachia* genera specifically characterized Ecuador populations (HU and AL, respectively). No specific genera were observed in the northern Peru population (PI). Additionally, *Corynebacterium* and *Micrococcus* (33.82 and 10.41%, respectively) were slightly more dominant bacterial genera in the southern Peru population (AY), while *Ochrobactrum* was the most frequently isolated from sand flies collected in the Ecuadorian Andes (61.46 and 67.22% for AL and HU, respectively) and northern Peru sites (PI, 66.16%) (Figure 3). As shown in Figure 3 and Figure S1, the microbial communities from Ecuador (AL, HU) and northern Peru (PI) were close to each other, while those from southern Peru (AY) were distant.

### 3.3. Fungal Profile of Field-Collected Lu. ayacuchensis

Fungi were only found in the southern Peru population (AY). Seven different genera were identified by homology to the ITS1 gene, with a few unclassified taxa at different ranks (Figure S2). These genera were not associated with phlebotomines except for the *Penicillium* genus. The genus *Papiliotrema* was more abundant than other genera, including *Penicillium*, *Exserohilum*, *Cladosprium*, *Sarocladium*, *Malassezia*, and *Debaryomyces* (99.79 and 0.21%, respectively) (Table S1).

## 4. Discussions

A previous study clearly demonstrated genetic divergence among populations of *Lu. ayacuchensis* with different vector competence, as indicated above, and its control has become a health priority [31]. Therefore, a complete analysis of the microbial community structure was performed in the present study to elucidate the role of the microbiota during the course of *L.* (*L.*) *mexicana* and *L. (V.) peruviana* infections by this important vector. The results obtained will provide critical information for the development of intervention strategies for sand fly control. Due to the difficulties associated with obtaining only the midgut of *Lu. ayacuchensis* since sand flies were collected for vector research and had to be identified at the species level, whole sand fly bodies from different areas in Peru and Ecuador were used in the microbiome analysis in the present study. However, a comparative analysis of microbial communities in whole bodies and midguts from our laboratory-reared *Phlebotomus papatasi* did not reveal any marked difference between samples. *Lu. ayacuchensis* populations collected from three distinct areas were used. Among the 21 dominant bacterial genera identified in the sand flies collected, 10 were detected for the first time, which may be due to limited information currently available on microbial diversity in sand flies. It is important to note that previous findings on gut bacterial communities in female sand flies from South America were obtained using classic techniques for molecular ecology, such as denaturing gradient gel electrophoresis (DGGE), except for a recent study in which a microbiome analysis of the digestive tracts of *Lu. evansi* adults was performed using 16S rRNA gene sequence amplicon high-throughput sequencing (Illumina MiSeq) [44]. The use of high-throughput technology enables a large number of reads in a single run, thereby providing a greater sampling depth than other techniques [45].

The present study revealed that *Actinobacteria* and *Proteobacteria* were the main phyla in all samples. These results are partly in agreement with the findings of a recent meta-analysis of sand fly-associated bacteria, which revealed that more than 57% and 47% of identified bacteria belonged to the *Proteobacteria* phylum in New and Old World sand fly species, respectively [46]. *Actinobacteria* showed lower abundance [11,44,47,48,49]. Previous studies reported that the phylum *Proteobacteria* contributed to host nutrition by fixing atmospheric nitrogen [50]. Due to the dominance of various taxa of *Actinobacteria* and *Proteobacteria* in sand flies, further studies are required to investigate their contributions to the life traits of sand flies.

A large fraction of genera were shared across all samples from the three different areas, such as the number of soil and environmental organisms including *Micrococcus* and *Corynebacterium* genera. However, the Ecuador and southern Peru populations each comprised individuals of particular genera. This result suggests that local soil and water environments play important roles in the colonization of sand flies by regional bacteria encountered at breeding sites or during sugar meals. Furthermore, sand flies collected from different sites of each population harbored similar microbes, suggesting a correlation between the sand fly-microbial association and the environment in which they reside. The present results also indicated that variations in the insect microbiota were influenced by the environment, similar to the type of feeding. Previous studies reported that variation in the microbiota residing in the insect gut might be mainly explained by the effects of the microhabitats, the gut morphology/structure, and physicochemical parameters, such as pH, oxygen availability in the insect gut, the type of diet, and host specificity [5,51]. On the other hand, based on preliminary results, the richness of the gut microbiota may be related to sand fly seasonal activity, and, thus, further investigations linked to seasonal microbiome variations in field sand flies are needed [46]. A microbiome study on sand fly species from the same areas is also required for the more detailed characterization of bacterial host specificity, which is an important component of vector competence affecting the nutritional and immune system status [52].

The 59 samples analyzed were screened for the presence of *Leishmania* spp. DNA and no positivity was found. These results are not surprising because the rate of *Leishmania* infection within sand flies is considered to be very low in nature, even in endemic areas [53]. As indicated above, *Lu. ayacuchensis* has contrasting susceptibility to *Leishmania* infection. The resident microbiota of sand flies clearly affects vector competence and may even partly explain the origin of these findings. The midgut is an important habitat for the development of *Leishmania* parasites, with the uninfective forms of *Leishmania* becoming infective. The bacterial composition of the sand fly gut has already been reported to affect physiological development [54] and immunity [55,56]. Among the bacterial genera associated with the *Lu. ayacuchensis* midgut, we identified pathogenic bacterial genera commonly found in the digestive tract of humans or other mammals, which have never been detected in the midguts of sand flies. Their occurrence in *Lu. ayacuchensis* midguts is insufficient to indicate sand flies as a possible vector but provides information on bacterial propagation by blood-feeding insects. Furthermore, it currently remains unclear whether certain clinical outcomes from leishmaniasis may be linked to bacteria deposited during a *Leishmania*-infected sand fly bite [57] because of natural selective pressure on some species of bacteria, *Leishmania* and their vectors are expected to influence the prevalence of *Leishmania* sp. infection directly.

Previous studies demonstrated that *Wolbachia* increases the resistance of arthropods to viruses [58,59,60] and/or alters their reproductive capacities [61,62]. *Wolbachia* was identified in 3 out of 9 samples collected from Ecuador (AL). In America, *Wolbachia* has been detected in *Lu. cruciata*, *Lu. shannoni*, *Lu. vespertilionis*, *Lu. trapidoi*, *Lu. longipalpis*, *Lu. whitmani*, *Lu. evansi*, and *Lu. intermedia* [11,44,63,64] and the present results expand our knowledge of its occurrence in *Lu. ayacuchensis*. As part of a paratransgenic approach, further studies are needed to evaluate its ability to colonize their gut and its interaction with *Leishmania* in various Ecuador samples (AL).

Only a few studies have attempted to identify fungal diversity in sand flies [12,20,21]. A high-throughput sequencing analysis revealed the absence of fungi in *Lu. longipalpis* guts collected from an endemic area, whereas fungi were found in a non-endemic area [20], suggesting that fungi are excluded in the presence of *Leishmania*. However, contradictory findings were obtained using culturing techniques that identified fungal genera in sand flies collected from endemic areas of northern Iran [21]. In the present study, fungal genera were identified in one of the endemic areas examined; however, it was not possible to elucidate their role or conclude any outcomes regarding potential pathogenic effects or interactions with *Leishmania*. A previous study reported a relationship between *L. (L.) infantum* infection in dogs without skin lesions and the increased growth of *Malassezia pachydermatis* with low phospholipase activity [65]. The first study of a *Malassezia* genus in *Lu. ayacuchensis* may pave the way for the functional characterization of sand fly-associated fungi.

## 5. Conclusions

The present study describes a complex microbial community structure in *Lu. ayacuchensis* with different vector competence collected from three distinct ecosystems in Ecuador and Peru. Sand flies have adapted to survive in diverse habitats, to feed on the blood of a range of hosts, and to transmit different *Leishmania* species. This ecological adaptation has an important impact on the bacterial composition of the gut, which may influence susceptibility to *Leishmania* infection. The results presented here will provide a baseline for understanding the role of the microbiota during the course of *L.* (*L.*) *mexicana* and *L.* (*V.*) *peruviana* infections in the important vector *Lu. ayacuchensis*. Any variations in midgut bacteria identified in the present study will need to be examined in more detail in future studies in order to robustly correlate gut microbiota differences with variations in *Leishmania* infectivity.

## Data Availability

All read sequences were deposited in the Sequence Read Archive (SRA) of NCBI under the accession numbers SAMN16706340-SAMN16706373 (https://www.ncbi.nlm.nih.gov/sra/PRJNA675377).

## Supplementary Materials

The following are available online at https://www.mdpi.com/2076-2607/9/1/68/s1, Figure S1: A heat map showing bacterial genera shared by two or more dominant sand flies, Figure S2: The fungal genus composition of the *Lutzomyia ayacuchensis* population collected from Ayacucho. Table S1: Sequence reads from each RNA sample collected from southern Peru.
