# Supplementary material for: Comparative Analysis of Bacterial Communities in Lutzomyia ayacuchensis Populations with Different Vector Competence to Leishmania Parasites in Ecuador and Peru
Source: Microorganisms. 2020 Dec 29;9(1):68. doi: 10.3390/microorganisms9010068 (PMC7823435; doi:10.3390/microorganisms9010068)
Supplement: Supplementary file 1 [file microorganisms-09-00068-s001.pdf]

Figure S1

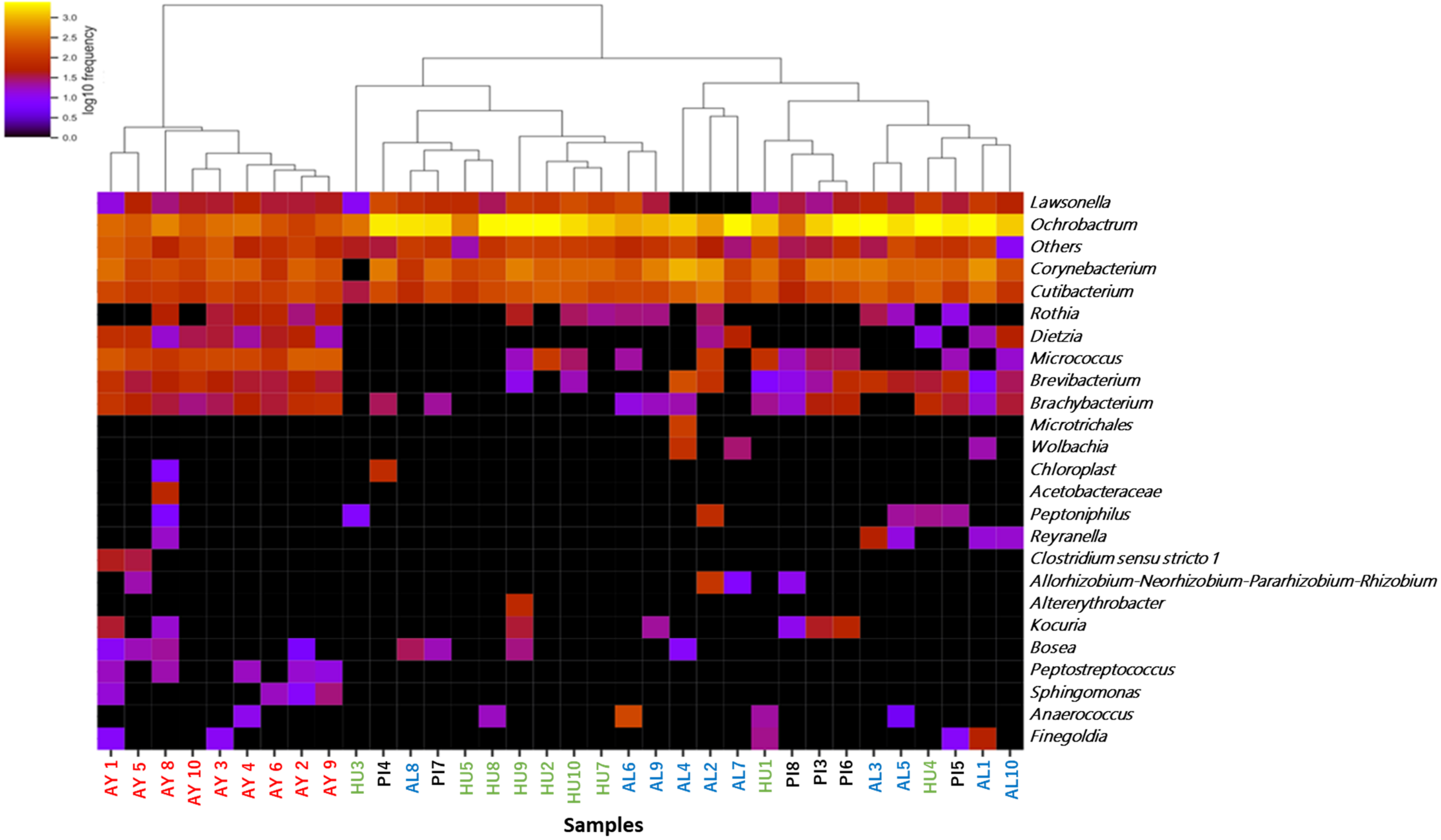

3.0
2.5
2.0
1.5
1.0
0.5
0.0
log10 frequency
Lawsonella
Ochrobactrum
Others
Corynebacterium
Cutibacterium
Rothia
Dietzia
Micrococcus
Brevibacterium
Brachybacterium
Microtrichales
Wolbachia
Chloroplast
Acetobacteraceae
Peptoniphilus
Reyranella
Clostridium sensu stricto 1
Allorhizobium-Neorhizobium-Pararhizobium-Rhizobium
Altererythrobacter
Kocuria
Bosea
Peptostreptococcus
Sphingomonas
Anaerococcus
Finegoldia
AY 1
AY 5
AY 8
AY 10
AY 3
AY 4
AY 6
AY 2
AY 9
HU3
PI4
AL8
PI7
HU5
HU8
HU9
HU2
HU10
HU7
AL6
AL9
AL4
AL2
AL7
HU1
PI8
PI3
PI6
AL3
AL5
HU4
PI5
AL1
AL10
Samples

Figure S2

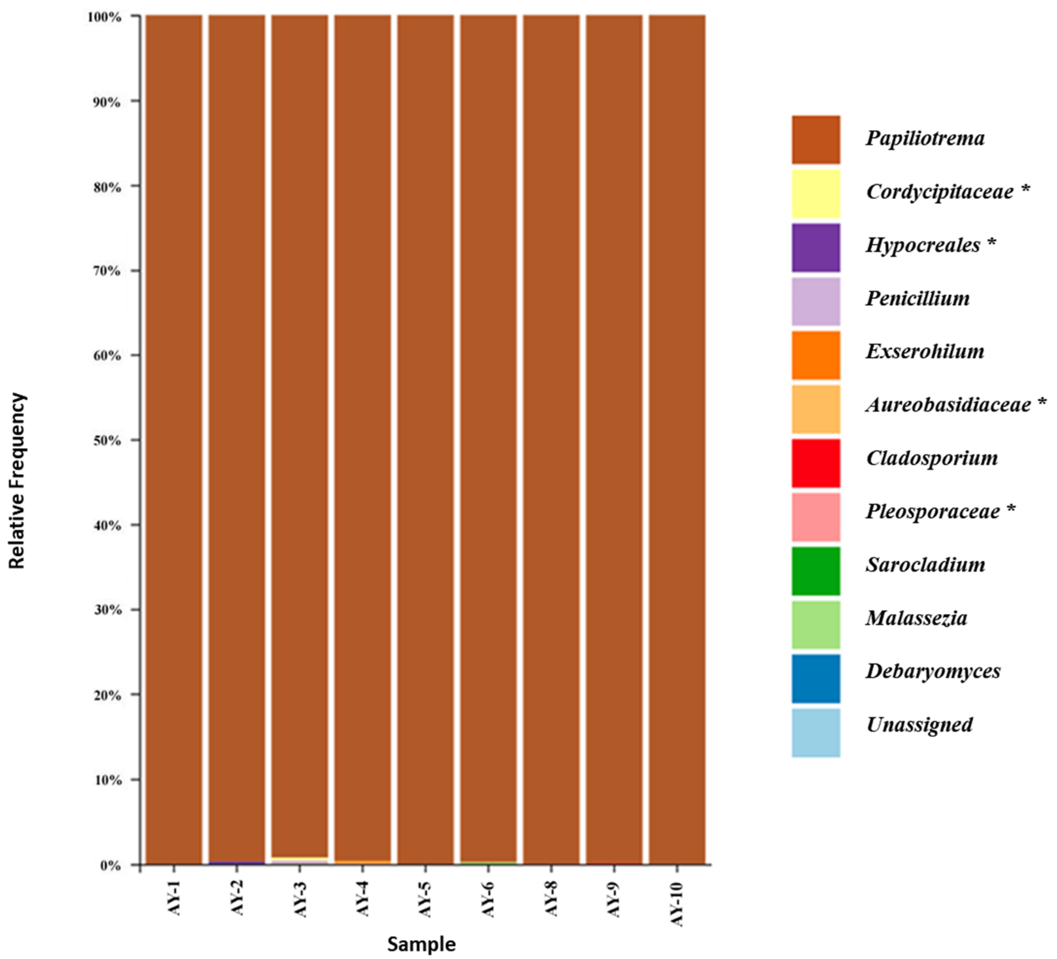

100%
90%
80%
70%
60%
50%
40%
30%
20%
10%
0%
Relative Frequency
AY-1
AY-2
AY-3
AY-4
AY-5
AY-6
AY-8
AY-9
AY-10
Sample
Papiliotrema
Cordycipitaceae *
Hypocreales *
Penicillium
Exserohilum
Aureobasidiaceae *
Cladosporium
Pleosporaceae *
Sarocladium
Malassezia
Debaryomyces
Unassigned

Table S1

Sequence reads from each RNA sample collected from southern Peru.

Genera	Southern Peru (AY) samples								
	AY1	AY2	AY3	AY4	AY5	AY6	AY8	AY9	AY10
Papiliotrema	172165	180639	185748	178744	204773	157314	180939	181648	220767
Penicillium	0	0	503	0	0	0	0	0	0
Exserohilum	0	0	0	358	0	0	0	0	0
Cladosporium	0	0	0	81	0	0	60	147	0
Sarocladium	0	0	0	0	0	177	0	0	0
Malassezia	0	0	62	0	0	0	0	0	20
Debaryomyces	0	0	69	0	0	0	0	0	0
**Others**	0	572	827	317	0	184	0	0	0
